# Phylogenomic Analyses of Echinodermata Support the Sister Groups of Asterozoa and Echinozoa

**DOI:** 10.1371/journal.pone.0119627

**Published:** 2015-03-20

**Authors:** Adrian Reich, Casey Dunn, Koji Akasaka, Gary Wessel

**Affiliations:** 1 Department of Molecular Biology, Cell Biology, and Biochemistry, Brown University, Providence, Rhode Island, United States of America; 2 Department of Ecology and Evolutionary Biology, Brown University, Providence, Rhode Island, United States of America; 3 Misaki Marine Biological Station, University of Tokyo, Miura, Japan; Laboratoire Arago, FRANCE

## Abstract

Echinoderms (sea urchins, sea stars, brittle stars, sea lilies and sea cucumbers) are a group of diverse organisms, second in number within deuterostome species to only the chordates. Echinoderms serve as excellent model systems for developmental biology due to their diverse developmental mechanisms, tractable laboratory use, and close phylogenetic distance to chordates. In addition, echinoderms are very well represented in the fossil record, including some larval features, making echinoderms a valuable system for studying evolutionary development. The internal relationships of Echinodermata have not been consistently supported across phylogenetic analyses, however, and this has hindered the study of other aspects of their biology. In order to test echinoderm phylogenetic relationships, we sequenced 23 *de novo* transcriptomes from all five clades of echinoderms. Using multiple phylogenetic methods at a variety of sampling depths we have constructed a well-supported phylogenetic tree of Echinodermata, including support for the sister groups of Asterozoa (sea stars and brittle stars) and Echinozoa (sea urchins and sea cucumbers). These results will help inform developmental and evolutionary studies specifically in echinoderms and deuterostomes in general.

## Introduction

Echinoderms are closely related to chordates and have been an invaluable model system for developmental biology for over one hundred fifty years [1,2]. Echinoderms are exclusively marine organisms, but they occupy many diverse benthic habitats, ranging from intertidal to deep sea. The adult body plan is unique among bilaterians as echinoderms demonstrate pentameric symmetry, while the larvae are bilaterally symmetrical. The pentameric adult rudiment forms within the developing, bilateral larva and upon metamorphosis, the bilateral larval structures typically are lost and yield a pentameric juvenile. This young adult develops by increasing its size, and internally, by embellishing its somatic gonad, within which the gametes form. Although a great number of morphological descriptions of this developmental process have been documented, the genes involved in development of the somatic gonad, and of the germ line in the echinoderm adults, are largely unknown. Further, the gonads contain a major representation of the nervous system and the paracrine system that regulates gonad function in response to changes in major environmental stimuli: diet, seasonality, and spawning.

The adult body of extant echinoderms is supported by a mesoderm-derived biomineralized skeleton that is calcareous, though evidence for magnesium carbonate skeletons exists in the fossil record [3]. The biomineralized skeleton can take many forms, from a single structure or test consisting of fused plates in the case of sea urchins and sand dollars, to segmented arms with a full range of motion in the case of sea stars and especially brittle stars. All echinoderms exhibit robust regenerative abilities, both as larvae and adults, though brittle stars and crinoids are especially adept at regeneration, especially in the adult [4–6]. Regeneration in the adults studied in echinoderms includes all major tissues; of particular note are the nervous system, gonads, and the germ line. Echinoderms have numerous advantages for the study of fertilization and early development (including external fertilization) [7] and significant effort has been invested in the understanding of the gene regulatory network (GRN) of the developing larvae of echinoderms, especially that of the purple sea urchin, *Stronglycentrotus purpuratus* [8].

**Fig 1:**
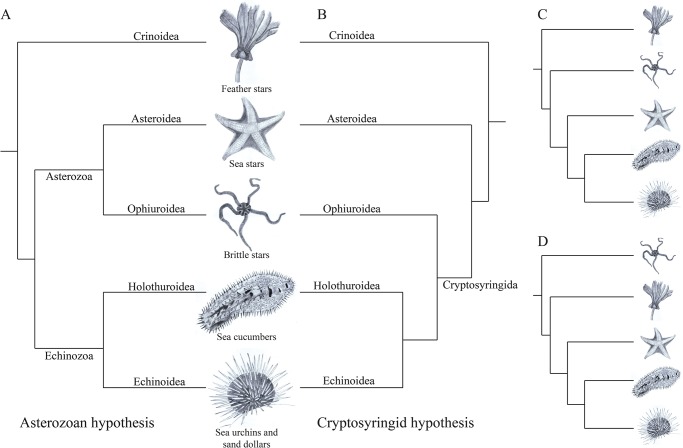
Four competing hypotheses of the phylogenetic relationship of extant echinoderms. Most phylogenetic studies place Echinoidea and Holothuroidea as sister groups. The difficulty lies in the placement of Ophiuroidea; different methods favor different positions for brittle stars. A) The predominant hypotheses of how extant echinoderms are related are the Asterozoan and Cryptosyringid hypotheses. B) Two other alternate hypotheses based on mitochondrial gene alignments.

Larvae of brittle stars and sea urchins in particular share many characters, including most strikingly a fenestrated larval skeleton which aids in the swimming and feeding of the pluteus larva. However, the phylogenetic relationships within Echinodermata have been controversial [9–13]. This is due, in part, to the very rapid radiation of Echinodermata that occurred within 10–15 million years after their most recent common ancestor more than 500 million years ago [14]. In recent years, four competing hypotheses have emerged, though many studies support either the Asterozoan or Cryptosyringid hypotheses (Fig. 1). The major difference between all hypotheses is the placement of the ophiuroids (brittle stars).

Morphological and embryological analyses support the Cryptosyringida hypothesis, [10,15] a monophyletic clade comprised of Echinoidea, Holothuroidea and Ophiuroidea (Fig. 1A). This was first formalized in name by Smith [9], but was originally proposed earlier [16]. In contrast, the Asterozoan hypothesis first proposed by Bather [17], which states that the Ophiuroidea is the sister group to Asteroidea, has been supported by some molecular sequence analyses [10,18]. A different approach tested if conservation of miRNAs was informative in phylogenomics analysis, but the study concluded that these small RNAs were uninformative for distinguishing between these hypotheses; although other molecular analyses within the study provided support for the Cryptosyringida hypothesis [12]. Analyses of mitochondrial genomes recovered two additional hypotheses for echinoderm relationships (Fig. 1B), placing Ophiuroidea as the sister group to all other echinoderms [19] or in a separate study, Asteroidea as sister to Echinozoa [13,20]. Here we revisit these open questions in echinoderm phylogeny with transcriptome data [21–23]. Our study differs from other recent phylogenetic analyses of transcriptomes [18,24] with respect to depth of sequencing, number of sampled taxa, and species analyzed. Our dataset comprises a 2 and 3 fold increase in the number of echinoderm taxa [18,24], respectively, and a 21 and 25 fold increase in aligned genes [18,24], respectively, which allows for the most thorough test yet of relationships within Echinodermata. We sequenced and assembled *de novo* transcriptomes of 23 different species of echinoderms, 18 of which had not been deeply sequenced before, and analyzed them in combination with 7 previously published datasets including 1 echinoderm (the purple sea urchin *S*. *purpuratus*) and 6 outgroups (*Aplysia californica*, *Branchiostoma floridae*, *Gallus gallus*, *Homo sapiens*, *Nematostella vectensis*, and *Saccoglossus kowalevskii*). Sequence assembly and annotation of the new data was performed with the software packaged Agalma, (an automated pipeline that assembles transcriptomes and constructs matrices for phylogenomic analysis) as was the identification of orthologous genes and matrix construction [23]. The scripts used to execute these analyses, as well as analysis reports, are available in a git repository [25]. We constructed two matrices that differed in gene sampling: a large, sparse matrix with 4,645 gene alignments with 34% gene occupancy, and a smaller, dense matrix with 1,125 gene alignments and 70% gene occupancy. Both were analyzed under the GAMMAWAG model with maximum likelihood (implemented in RAxML), and the dense matrix was also analyzed with bayesian methods (implemented in PhyloBayes) under the CAT-GTR model. These three phylogenetic analyses are the focus of the results presented here.

## Materials and Methods

### RNA isolation and sequencing

Whole ovary was dissected from gravid females and placed in Trizol (Invitrogen). RNA was extracted and then cleaned with a Qiagen RNeasy Micro column with on-column DNA digestion. The sequencing libraries were prepared with Illumina reagents, (mRNA-Seq Sample Prep Kit for GAIIx samples or TruSeq Sample Prep Kit for HiSeq samples) with the maximum recommended RNA input. The protocol was followed exactly with the addition of a gel selection step of 400–500bp, (agarose gel for GAIIx samples or Caliper LabChip XT for HiSeq samples) prior to PCR amplification.

### Transcriptome assemblies and RefSeq data

The reads documented in S1 Table were processed and assembled using the agalma pipeline (ver. 0.3.5, https://bitbucket.org/caseywdunn/agalma) [23,26] which wraps the trinity *de novo* transcriptome assembler (ver. r2013_08_14) [27,28]. The default settings were used within agalma to assemble all transcriptomes. A total of three echinoderm public datasets were also *de novo* assembled and used in this analysis. One dataset consisting of combined 2 day (SRR496203) and 6 day (SRR496204) old larvae of *Parastichopus parvimensis* [29] was assembled as above. Two additional brittle star 454 datasets were assembled using newbler (DataAnalysis ver. 2.9, Roche). Reads isolated from regenerating arms of *Ophionotus victoriae* [4] (SRR500294) were assembled as follows: “runAssembly-cdna”; reads from gastrula larvae of *Ophiocoma wendtii* [30] were assembled as follows: “runAssembly-cpu 8-vt Adapters.fasta-cdna" (for Adapters.fasta, see S2 Table). Exemplar transcripts were then selected from the assembled 454 datasets as previously described [31] and the script has been deposited in the github repository [25]. Seven RefSeq datasets were downloaded: *Aplysia californica*, *Branchiostoma floridae*, *Gallus gallus*, *Homo sapiens*, *Nematostella vectensis*, *Saccoglossus kowalevskii*, and *Strongylocentrotus purpuratus*. All except for the human dataset were from RefSeq release 62, dated Nov. 10, 2013; the human dataset was downloaded Dec. 9, 2013.

### Post assembly and phylogenetic analyses

All 30 datasets (23 de novo assembled transcriptomes and 7 RefSeq datasets; S1 Table) were compared against NCBI SwissProt using BLASTx with a cutoff of 0.000001 [23]. TransDecoder was used to translate sequences, and similar translated sequences were identified by a pair wise BLASTp followed by clustering using MCL [32] to identify orthologous genes. Two supermatricies that differ in gene sampling were used in the phylogenetic analyses: ‘sparse’ and ‘dense’. The ‘sparse’ supermatrix has all 30 taxa and 34% matrix occupancy, (S4A Fig.) with 4,645 peptide sequences and 630,945 amino acid sites. The ‘dense’ supermatrix is a subset of the sparse matrix. It has all 30 taxa and 70% matrix occupancy, (S4B Fig.) with 1,125 peptide sequences and 101,652 amino acid sites. Maximum likelihood phylogenetic analyses were done with RAxML (GAMMAWAG model) with 1,000 boot strap iterations on the ‘dense’ supermatrix, and using the same model, 100 boot strap iterations on the ‘sparse’ supermatrix. Both supermatrices and calculated phylograms can be downloaded from the Data Dryad Repository [33].

Baysian phylogenetic analyses were done with PhyloBayes 1.3b-mpi [34] on the ‘dense’ supermatrix using the CAT-GTR model with the following command: “pb_mpi-S-d supermatrix.dense.phylip-cat-gtr outputFile”. A total of 31,623 generations were run over three chains. All three chains converged within 2,000 generations (S5 Fig.) and after removing these 2,000 from each chain and sampling every 10 trees, the maximum difference was 1.17×10^-3^, (2,561 sampled trees), and a majority consensus tree was constructed.

The SOWH test was implemented using the sowhat software package, [35] (https://github.com/josephryan/sowhat) with the following command: “perl sowhat—constraint = Alternate.hypo.tre—aln = supermatrix.dense.phylip—model = PROTGAMMAWAG—dir = outDir—name = outFile—rax = 'raxmlHPC-PTHREADS-SSE3-T 16'—seqgen = seq-gen—reps = 500—stop >output.txt”. Three alternate hypothesis trees were constructed to test: A) the cryptosyringid hypothesis, B) Ophiuroidea sister to the rest of echinoderms, and C) Asteroidea sister to Echinozoa (S3 Fig.). All three analyses were stopped after at least 99 iterations because any additional sampling was very unlikely to change the results (S3 Fig.).

### Data availability

The raw reads and assembled trasncriptomes reported in this paper have been deposited in the GenBank database (NCBI BioProject no. PRJNA236087). Assembly statistics, agalma resource reports and scripts used in the analyses can be found in the git repository [25] and the supermatrix alignments and all trees including SOWH constraint trees can be downloaded from the Dryad Digital Repository [33]. All *de novo* transcriptomes can also be accessed on echinobase.org, including BLAST searches of the annotated transcripts.

## Results and Discussion

The assembly of the *de novo* Illumina transcriptomes yielded on average 24,033 high quality contigs with a match to SwissProt (S1 Table). The number of transcripts with SwissProt hits in the *de novo* transcriptomes was comparable to the number of sequences in the RefSeq datasets we included for other taxa (S1A Fig.). The number of SwissProt sequences did not appear to be artificially inflated by fragmented assemblies (S1B Fig.). The comparable number of SwissProt sequences and comparable size of the N50s of the *de novo* transcriptomes in relation to the RefSeq datasets both suggest that the assembled transcriptomes are of high quality; especially compared with the *S*. *purpuratus* RefSeq dataset, an echinoderm with a well sequenced and annotated genome [8].

**Fig 2:**
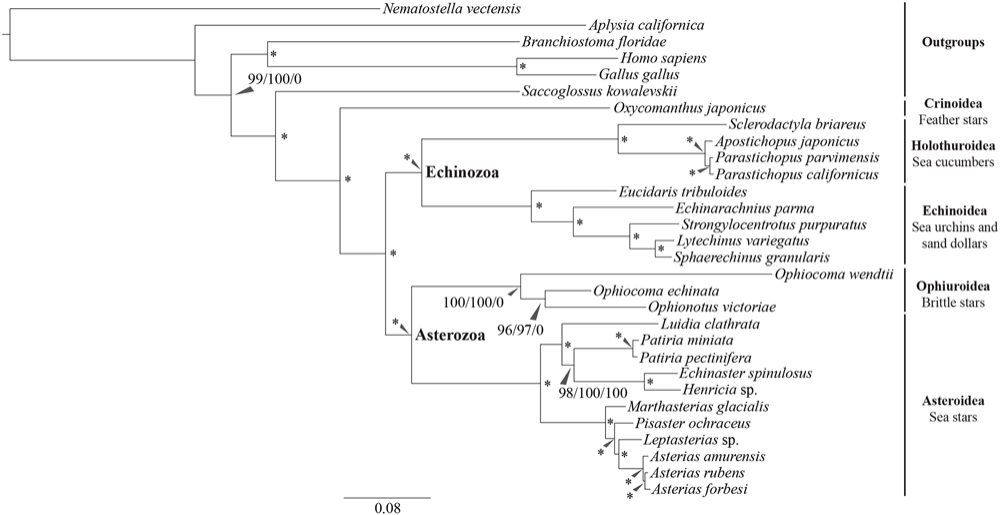
Phylogenetic relationship of extant echinoderms. Support values for the phylogenetic trees using RAxML and PhyloBayes on the dense and sparse supermatricies. Each node is scored with three support values; an asterisk denotes 100/100/100 support. The first support value is the dense supermatrix RAxML 1,000 bootstraps, the second value is the sparse supermatrix RAxML 100 bootstraps, and the third value is the dense supermatrix PhyloBayes posterior probabilities. The phylogram presented here is from the dense supermatrix RAxML analysis. See S2 Fig. for the tree topology predicted by the PhyloBayes analysis.

All phylogenetic analyses (with different sampling depth, aligned genes, models of molecular evolution, phylogenetic inference programs and matrix occupancy) placed Ophiuroidea as the sister group to Asteroidea (Figs. 1, 2, and S2 Fig.), consistent with the Asterozoan hypothesis of echinoderm evolution. We find no support for alternative hypotheses of echinoderm phylogeny, such as the placement of Ophiuroidea as the sister group to all other echinoderms [19] or as the sister group to Echinozoa (i.e., the Cryptosyringida hypothesis) [9,12,15]. This is consistent with a recent transcriptome analysis based on a smaller number of genes and taxa [18]. In that study, Telford *et al*. ran separate analyses on different subsets of genes based on the ranking of how quickly the genes evolved which generated different results. In our dataset, due to the very high occupancy in the dense supermatrix (70%), it is unlikely to include many quickly evolving genes, especially since at least 5% of the unoccupied space in the supermatrix is confined to the two poorly sampled taxa (S4B Fig.).

In order to test further the placement of brittle stars as sister group to sea stars and not a member of Cryptosyringida (Fig. 1), we performed the Swofford-Olsen-Waddell-Hillis (SOWH) test [36] using the RAxML tree constructed from the dense supermatrix. We used the SOWH test rather than the Kishino-Hasegawa (KH) [37] test, which is appropriate to use when comparing two *a priori* hypotheses [38]. In order to compare one *a priori* tree with the maximum likelihood RAxML tree generated from this dataset, the parametric SOWH can avoid any selection bias and is more appropriate [38]. We constructed a tree with all hypothesized members of Cryptosyringida (Fig. 1) as a polytomy, which was sister to a second polytomy containing the remaining taxa, in order to specifically test the Cryptosyringid hypothesis. This *a priori* tree was used as the constraint for which we found no support (*p*-value = 0, S3A Fig.). These results strongly reject this alternate hypothesis. Two additional SOWH tests were conducted to test the hypothesis that Ophiuroidea is sister to the rest of echinoderms [19] or that Asteroidea is sister to Echinozoa [13,20]; we found no support for either (*p*-values = 0, S3B,C Fig.). The means, standard errors, variances, and standard deviations of the null distribution for all three analyses were all less than 3.22×10^-6^. Furthermore, the difference between the log-likelihood of the unconstrained RAxML tree and the log-likelihood of the constrained trees for the hypotheses Cryptosyringida, Asteroidea sister to Echinozoa, and Ophiuroidea sister to echinoderms was 444.98, 323.60, and 1,286.02, respectively, resulting in *p*-values of 0 for all three analyses. The SOWH test uses the constraint tree to simulate the evolution of the data assuming that the *a priori* tree is correct. It then calculates the maximum likelihood of this tree and compares it with the maximum likelihood of the hypothesized tree; because the difference is so large for all three constraint trees, we can confidently reject the *a priori* trees in favor of the Asterozoa hypothesis.

The strong support for the Asterozoa hypothesis of echinoderm phylogeny found here impacts our interpretation of the origin of multiple characters in these animals. This includes, for example, the origin of a skeleton in larvae. A larval skeleton is present in both Ophiuroidea and Echinoidia, but not in the Holothuroidea. Were the Cryptosyringid hypothesis to be correct, we would posit that the larval skeleton was “simply” lost in Holothuroidea. This would be a parsimonious explanation of the presence and absence of such a trait in this relationship, but is now also inconsistent with results obtained here. Recent experimental analyses of mesodermal gene regulation in a sea cucumber documents that only a rudimentary regulatory state appears to exist in an extant sea cucumber (*Parastichopus parvimensis*), concluding that the common ancestor of the sea cucumber and the sea urchin, at best, had an undeveloped larval skeleton [39]. The premise of a plesiomorphic larval skeleton is also not compatible with the conclusion that transference of the adult skeleton gene regulatory network into the larval stage was a relatively simply reutilization of existing adult circuitry. All classes of echinoderms have an adult skeleton, and since the larval skeleton does not appear to be a shared ancient character of a Cryptosyringid clade, we instead acknowledge and invoke the likely transference of the adult skeletal machinery into the larvae of the Ophiuroidea and Echinoidia, but not in Crinodea, Asteroidea, and Holothuroidea. We feel this is the most parsimonious explanation of the origin of larval skeletons, given the new support for echinoderm phylogenomics reported here.

Asteroids are often used in the study of meiotic events. These animals harbor large oocytes, all stored fully grown in the ovary with their germinal vesicles intact. Upon spawning, the oocytes are induced to reinitiate meiotic divisions, and are often completed only after fertilization [7]. Since the trigger for meiotic resumption is well known to be 1-methyl-adenine [40], and the animal stores upwards of several million oocytes, investigators use this cell frequently for meiotic studies. Echinoids, on the other hand, are the only deuterostome clade known to store their eggs already having completed meiosis. This difference in oocyte maturation and fertilization greatly impacts the fertilization characteristics of course, but also likely the developmental strategy of the organism. The echinoids are also the only taxon with unequal cleavages early in development, that segregate their germ line within the first five cell divisions, and that have segregated all meiotic events from the rapid mitotic events of early development. Now gaining confidence in the evolutionary relationships of these organisms, we conclude that the major transitions in echinoids occurred following the split between Asterozoa and Echinozoa. Since all the sequence analysis from this study was performed on tissue-matched samples of ovaries, which contain developing oocytes, we will not be focusing on Cryptosyringida as the last common ancestor for these transitions, and instead, will be honing comparisons within Holothuroidea. Additionally, the ovarian sequences presented here will be a rich source to test for speciation and reproductive isolation. Effective analysis of the sequences undergoing positive selection may reveal candidates for sperm interaction or support for different reproductive strategies [41].

All other deep nodes in the echinoderm phylogeny also had strong support across analyses, providing the most rigorous test yet of these relationships. Our analyses also provide insight into the relationships within some echinoderm subclades. A particularly interesting result is the internal phylogenetic relationships of the sea stars, which differ from a number of previous analyses [42–46]. We found evidence against Valvatacea as the three sampled species in this group (*Luidia clathrata*, *Patiria pectinifera*, *Patiria miniata*) are paraphyletic relative to Spinulosacea (here represented by *Henricia sp* and *Echinaster spinulosus*). All our analyses support *Pisaster* as sister group to the clade comprised of *Asterias* and *Leptasterias* (Fig. 2). The largest difference between many of the previous studies of asteroidean phylogeny and the current results was the placement of Paxillosida (represented in this study by *Luidia clathrata*). In the analyses reported here, Paxillosida was sister to Valvatida (*Patiria miniata*) and Spinulosida, which were in turn sister to each other (Fig. 2). This is in contrast to the previous placement of Paxillosida as a sister group to Forcipulatida [44], sister to all of Asteroidea [42], or branching much later in the evolution of Asteroidea [46]. The placement of Paxillosida presents several interesting hypotheses considering the lack of an anus and the suckers on tube feet in Paxillosida [47,48]. Either an anus and suckers were present in the most recent common ancestor of Asteroidea and then secondarily lost in Paxillosida, or these features arose independently in Forcipulatida and the clade comprised of Valvatida and Spinulosida.

The size of data matricies and their occupancy has varied considerably over recent phylogenomic studies, which have differed substantially in the sequencing technologies used as well as methods for identification of orthologous genes and matrix construction. The present study uses a similar matrix construction technique as previous analyses [31], but the occupancy of the matrix is much higher. This suggests that at least part of the reason for reduced occupancy in some previous analyses was due to data acquisition, and that future studies based entirely on the most recent sequencing technologies will have much improved gene sampling.

Overall, the present study provides the greatest depth and breadth of new sequence analysis for consideration of the phylogenetic relationships of echinoderms. Coupled with the recent study using 219 genes for analysis from four newly sequenced echinoderms [18], and of a broader analysis of ambulacraria using 185 genes, including six newly sequenced echinoderms [24], mostly non-overlapping tissues and animals from the present work, we now have a rich analysis to present. Since each these studies used different animals, different methodology of analysis, and different depths of data consideration, yet they each came to the same conclusions in regards to the major echinoderm taxon relationships, we now have great confidence in the relationships of these animals and provide deep datasets for further analysis.

## Supporting Information

S1 Fig Histograms of the postassembly comparisons of RefSeq and *de novo* assembled datasets. **(A)** The numbers of SwissProt transcripts is comparable between RefSeq datasets (green and purple) and *de novo* assembled transcriptomes (orange). **(B)** Comparing the N50 of the SwissProt transcripts, the *de novo* transcriptomes are on average only slightly smaller than the RefSeq datasets. The *S*. *purpuratus* RefSeq dataset is in purple, outgroup RefSeq datasets in green and *de novo* assembled transcriptomes in orange; colours as in S1 Table. The number of transcriptomes is on the ordinate axis.
(TIF)

S2 Fig Phylogenetic relationship of extant echinoderms. Support values for the phylogenetic trees using RAxML and PhyloBayes on the dense and sparse supermatricies. Each node is scored with three support values; an asterisk denotes 100/100/100 support. The first support value is the dense supermatrix RAxML 1,000 bootstraps, the second value is the sparse supermatrix RAxML 100 bootstraps, and the third value is the dense supermatrix PhyloBayes posterior probabilities. The phylogram presented here is from the dense supermatrix PhyloBayes analysis.
(TIF)

S3 Fig Cumulative null distributions of SOWH test iterations. The SOWH tests were run for at least 99 iterations on the dense supermatrix and were stopped because any further iterations were unlikely to change. The three SOWH tests used the following tree topologies to test: (A) the crytosynirgid hypothesis, ((sea urchins, sea cucumbers, brittle stars),(sea stars, feather star, outgroups));, (B) Ophiuroidea sister to the rest of echinoderms, ((sea urchins, sea cucumbers, sea stars, feather star),(brittle stars, outgroups));, and (C) Asteroidea sister to Echinozoa ((sea urchins, sea cucumbers, sea stars),(brittle stars, feather star, outgroups));. None of the three tests found support for any alternate hypothesis with *p*-values all equal to 0.
(TIF)

S4 Fig Visual representation of the sparse and dense supermatricies including all thirty taxa. Each horizontal row is a single taxa and each vertical column is a gene alignment; presence is marked in black and absence in white. The taxa are arranged from top to bottom from most genes present to least. (A) The sparse supermatrix is 34% occupied, contains all 30 taxa, and contains alignments of 4,645 peptide sequences. (B) The dense supermatrix is 70% occupied, contains all 30 taxa, and contains alignments of 1,125 peptide sequences.
(TIF)

S5 Fig Test of convergence of PhyloBayes chains. All three independent chains converge with a maximum difference of 1.17×10^-3^ after a burn-in of 2000 generations (dashed line).
(TIF)

S1 Table Assembly and post assembly summary by taxa. Public datasets (red background) were obtained from RefSeq, the SRA and personal communication; new datasets from this study are in blue. Data from RefSeq (green and purple background) were compared with the *de novo* transcriptomes assembled in this study (orange background). Members of Echinodermata are in purple or orange and organisms serving as phylogenetic outgroups are in green. The Sample Source denotes public data (RefSeq), sequence data from Roche (454), and paired-end (PE) sequence data from Illumina of a given length (72bp, 80bp, or 100bp per read).
(PDF)

S2 Table High copy sequences trimmed from *O*. *wendtii* 454 assembly. Sequences trimmed from individual 454 reads during the assembly of the *O*. *wendtii* transcriptome. Some sequences are technical in nature and should be removed (e.g. adapters), though other sequences were removed simply due to their very high abundance. Highly repeated sequences greatly increase the computational burden of the assembly and were therefore removed.
(PDF)
