# Supplementary figures and images for: Phylogenomic Analyses of Echinodermata Support the Sister Groups of Asterozoa and Echinozoa
Source: PLoS One. 2015 Mar 20;10(3):e0119627. doi: 10.1371/journal.pone.0119627 (PMC4368666; doi:10.1371/journal.pone.0119627)

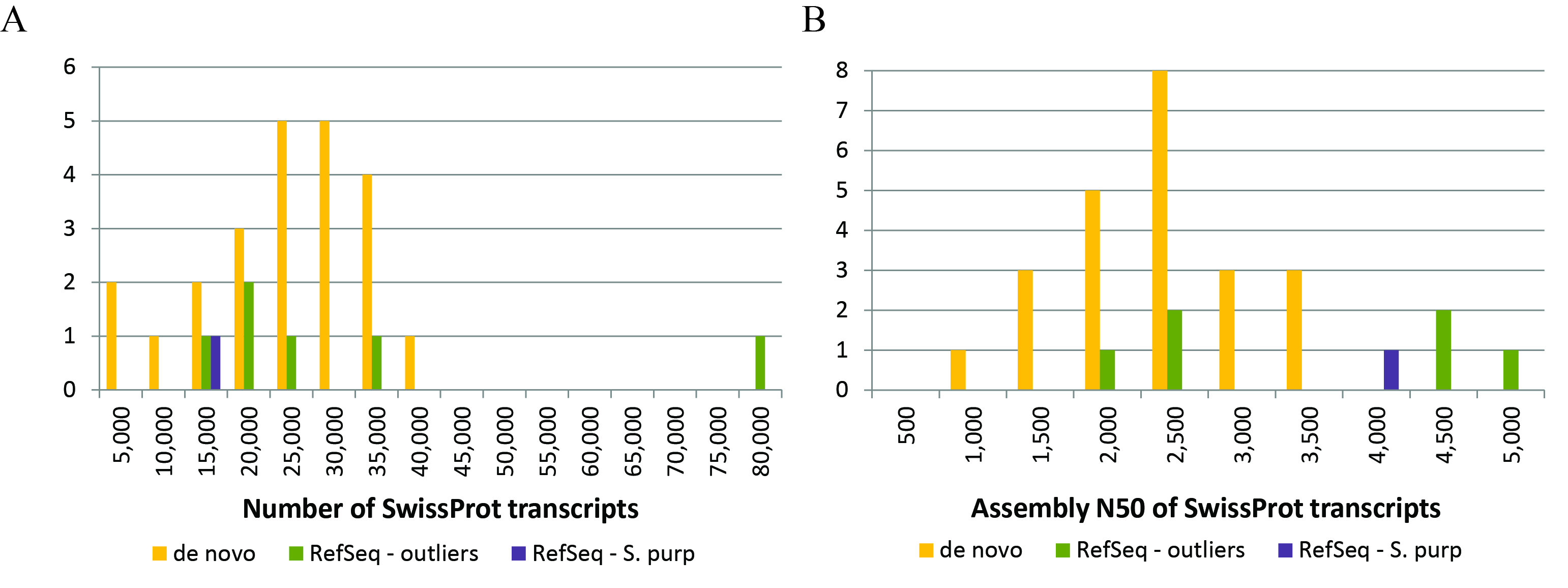

Supplement: S1 Fig — (A) The numbers of SwissProt transcripts is comparable between RefSeq datasets (green and purple) and de novo assembled transcriptomes (orange). (B) Comparing the N50 of the SwissProt transcripts, the de novo transcriptomes are on average only slightly smaller than the RefSeq datasets. The S. purpuratus RefSeq dataset is in purple, outgroup RefSeq datasets in green and de novo assembled transcriptomes in orange; colours as in S1 Table. The number of transcriptomes is on the ordinate axis. (TIF) [file pone.0119627.s001.tif]

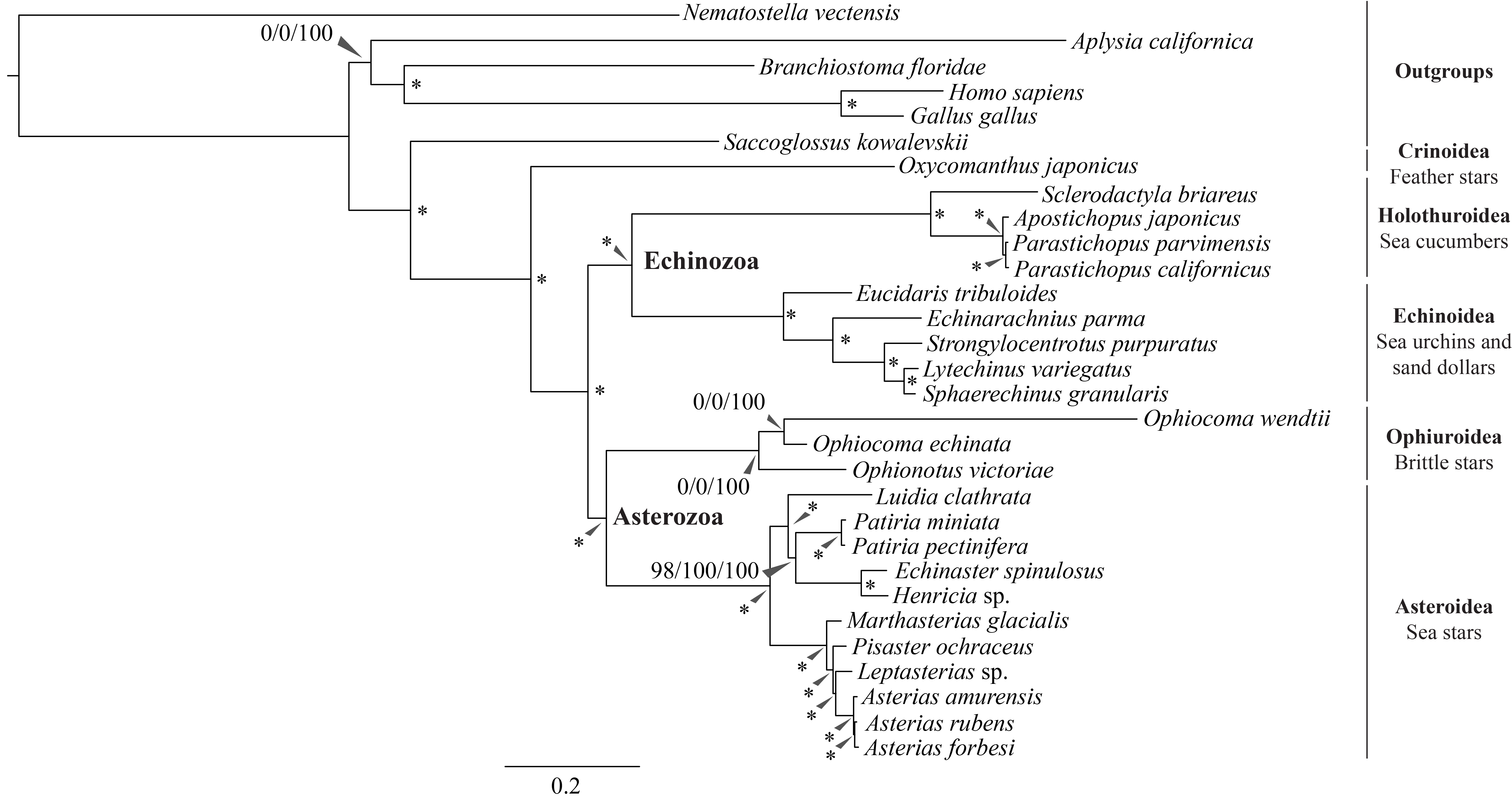

Supplement: S2 Fig — Support values for the phylogenetic trees using RAxML and PhyloBayes on the dense and sparse supermatricies. Each node is scored with three support values; an asterisk denotes 100/100/100 support. The first support value is the dense supermatrix RAxML 1,000 bootstraps, the second value is the sparse supermatrix RAxML 100 bootstraps, and the third value is the dense supermatrix PhyloBayes posterior probabilities. The phylogram presented here is from the dense supermatrix PhyloBayes analysis. (TIF) [file pone.0119627.s002.tif]

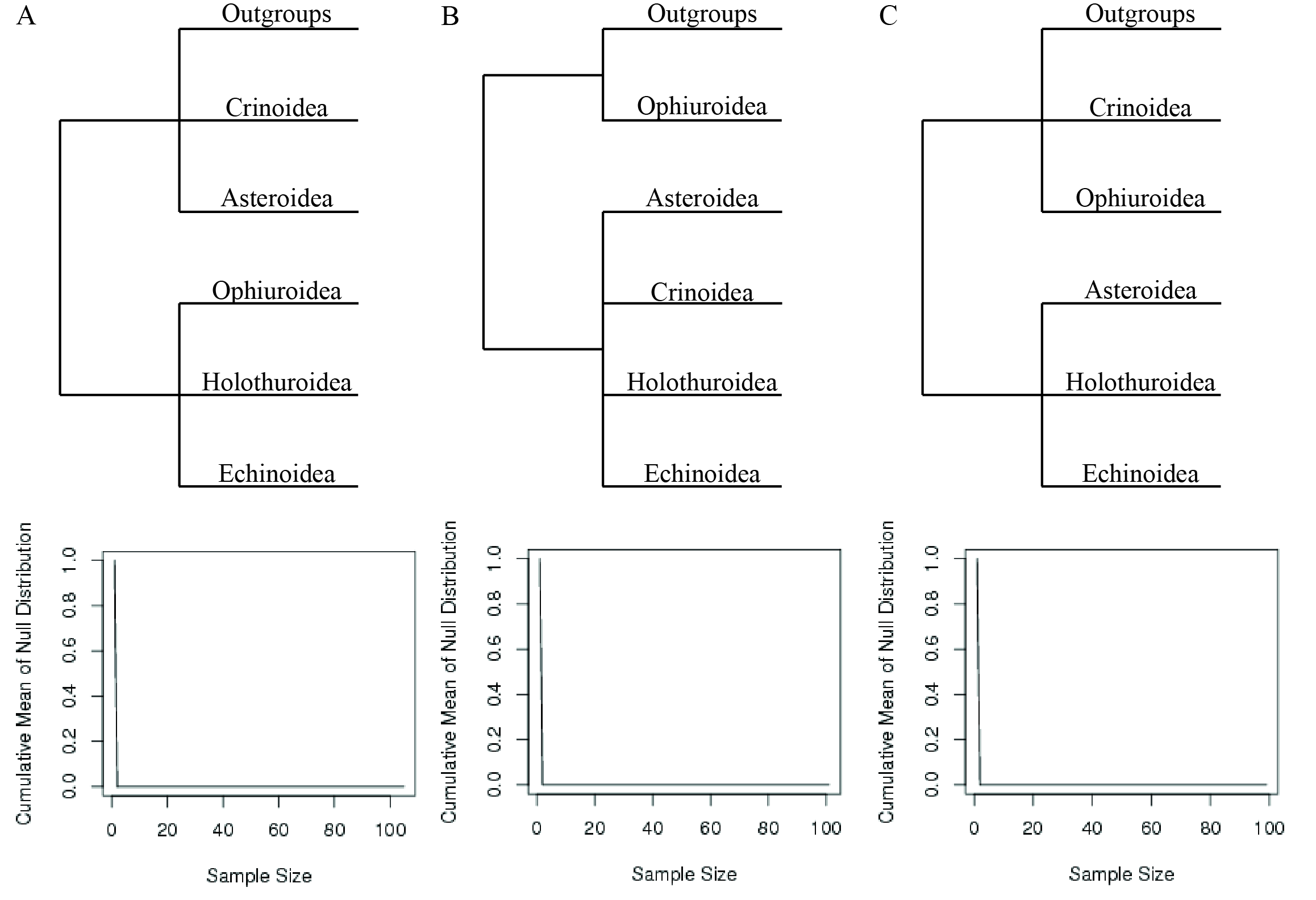

Supplement: S3 Fig — The SOWH tests were run for at least 99 iterations on the dense supermatrix and were stopped because any further iterations were unlikely to change. The three SOWH tests used the following tree topologies to test: (A) the crytosynirgid hypothesis, ((sea urchins, sea cucumbers, brittle stars),(sea stars, feather star, outgroups));, (B) Ophiuroidea sister to the rest of echinoderms, ((sea urchins, sea cucumbers, sea stars, feather star),(brittle stars, outgroups));, and (C) Asteroidea sister to Echinozoa ((sea urchins, sea cucumbers, sea stars),(brittle stars, feather star, outgroups));. None of the three tests found support for any alternate hypothesis with p-values all equal to 0. (TIF) [file pone.0119627.s003.tif]

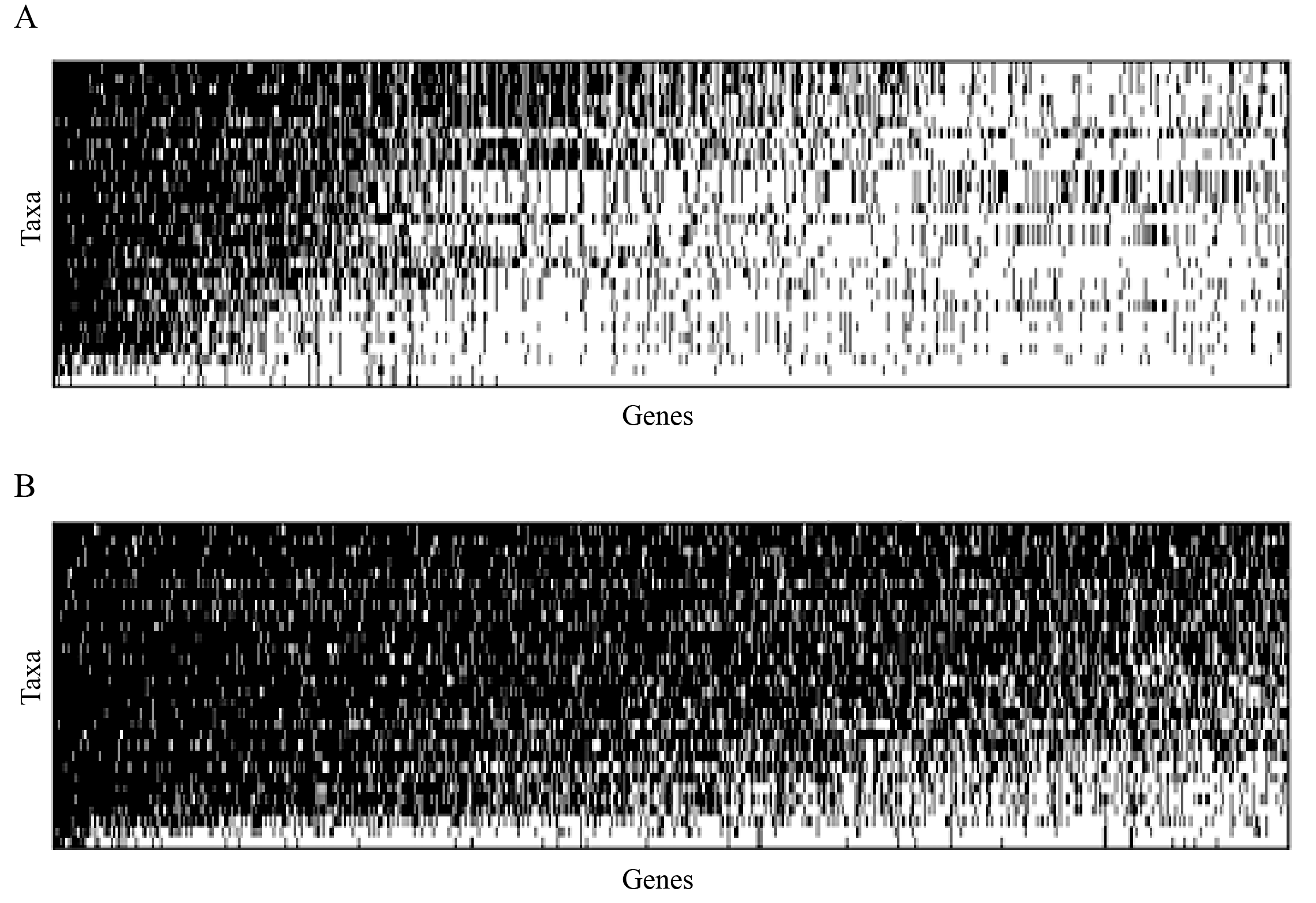

Supplement: S4 Fig — Each horizontal row is a single taxa and each vertical column is a gene alignment; presence is marked in black and absence in white. The taxa are arranged from top to bottom from most genes present to least. (A) The sparse supermatrix is 34% occupied, contains all 30 taxa, and contains alignments of 4,645 peptide sequences. (B) The dense supermatrix is 70% occupied, contains all 30 taxa, and contains alignments of 1,125 peptide sequences. (TIF) [file pone.0119627.s004.tif]

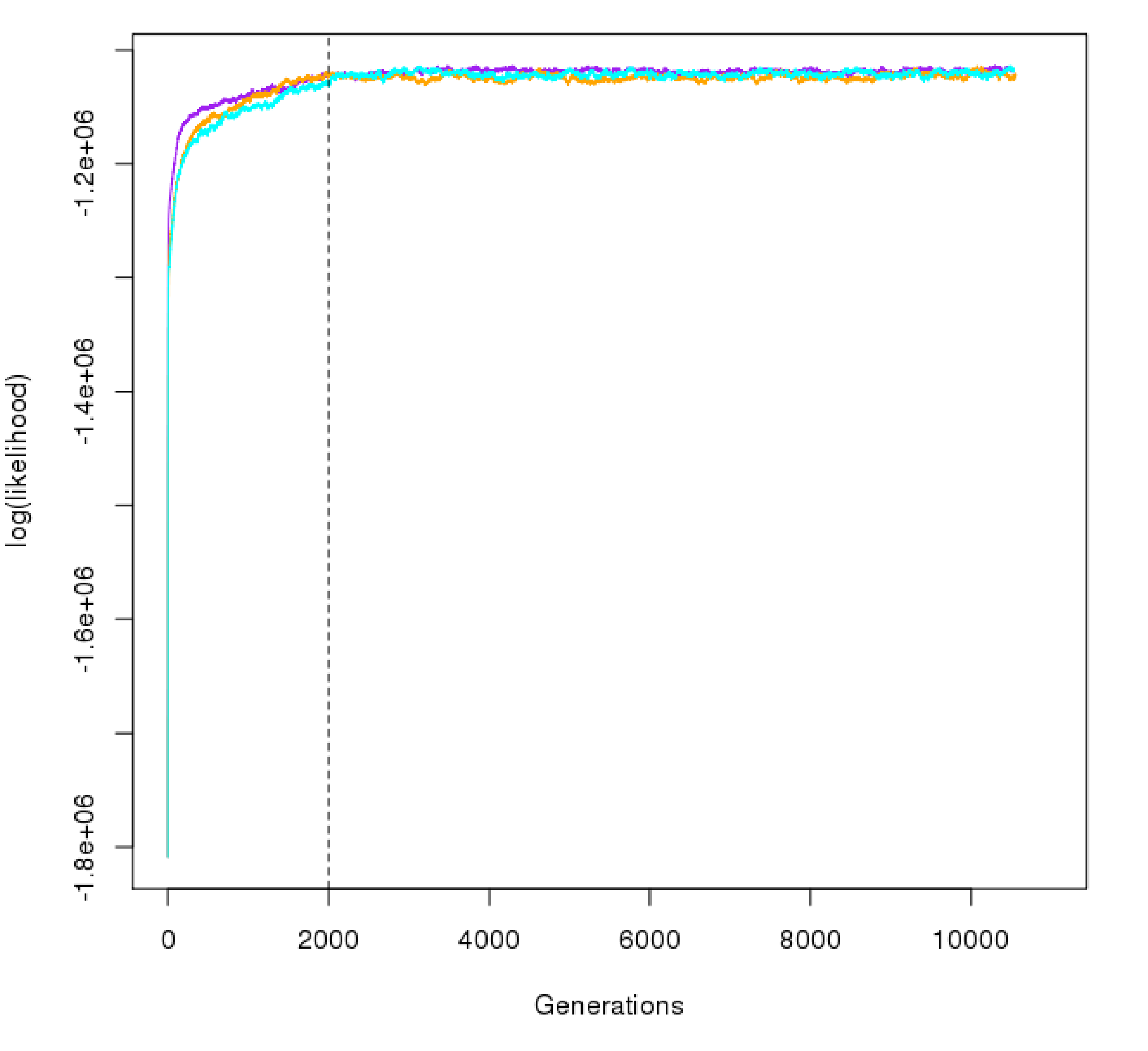

Supplement: S5 Fig — All three independent chains converge with a maximum difference of 1.17×10^-3^ after a burn-in of 2000 generations (dashed line). (TIF) [file pone.0119627.s005.tif]
