# Supplementary material for: Phylogenomic Analyses of Echinodermata Support the Sister Groups of Asterozoa and Echinozoa
Source: PLoS One. 2015 Mar 20;10(3):e0119627. doi: 10.1371/journal.pone.0119627 (PMC4368666; doi:10.1371/journal.pone.0119627)
Supplement: S1 Table — Public datasets (red background) were obtained from RefSeq, the SRA and personal communication; new datasets from this study are in blue. Data from RefSeq (green and purple background) were compared with the de novo transcriptomes assembled in this study (orange background). Members of Echinodermata are in purple or orange and organisms serving as phylogenetic outgroups are in green. The Sample Source denotes public data (RefSeq), sequence data from Roche (454), and paired-end (PE) sequence data from Illumina of a given length (72bp, 80bp, or 100bp per read). (PDF) [file pone.0119627.s006.pdf]

Species	Assembled Transcripts	Sample Source	SwissProt Transcripts	SwissProt N50
Aplysia californica	26,249	RefSeq	18,424	4,099
Branchiostoma floridae	28,575	RefSeq	21,881	2,127
Gallus gallus	36,995	RefSeq	31,868	4,527
Homo sapiens	91,944	RefSeq	77,696	4,208
Nematostella vectensis	24,462	RefSeq	18,261	1,609
Saccoglossus kowalevskii	12,851	RefSeq	10,936	2,294
Strongylocentrotus purpuratus	23,078	RefSeq	19,554	4,363
Ophiocoma wendtii	5,025	454	1,995	771
Ophionotus victoriae	3,210	454	694	1,645
Parastichopus parvimensis	107,585	72bp PE	34,809	3,076
Eucidaris tribuloides	45,385	80bp PE	7,410	1,307
Patiria miniata	76,847	80bp PE	24,679	2,494
Oxycomanthus japonicus	39,225	80bp PE	15,868	2,816
Ophiocoma echinata	111,491	80bp PE	21,485	2,454
Lytechinus variegatus	90,621	80bp PE	25,994	2,914
Asterias forbesi	68,714	80bp PE	22,625	2,784
Sclerodactyla briareus	58,273	80bp PE	19,297	3,154
Asterias rubens	81,470	100bp PE	25,733	2,349
Henricia species	137,160	100bp PE	36,754	2,231
Echinaster spinulosus	119,580	100bp PE	34,231	2,314
Echinarachnius parma	96,977	100bp PE	26,016	2,017
Leptasterias species	108,544	100bp PE	33,803	3,172
Luidia clathrata	84,380	100bp PE	23,407	1,835
Marthasterias glacialis	118,847	100bp PE	28,327	1,928
Pisaster ochraceus	37,111	100bp PE	10,361	1,225
Parastichopus californicus	30,607	100bp PE	10,379	1,132
Sphaerechinus granularis	92,460	100bp PE	24,024	2,008
Apostichopus japonicus	85,061	100bp PE	26,902	1,943
Patiria pectinifera	118,294	100bp PE	33,009	1,746
Asterias amurensis	63,300	100bp PE	19,592	2,088
