# Supplementary material for: Phylogenomic Analyses of Echinodermata Support the Sister Groups of Asterozoa and Echinozoa
Source: PLoS One. 2015 Mar 20;10(3):e0119627. doi: 10.1371/journal.pone.0119627 (PMC4368666; doi:10.1371/journal.pone.0119627)
Supplement: S2 Table — Sequences trimmed from individual 454 reads during the assembly of the O. wendtii transcriptome. Some sequences are technical in nature and should be removed (e.g. adapters), though other sequences were removed simply due to their very high abundance. Highly repeated sequences greatly increase the computational burden of the assembly and were therefore removed. (PDF) [file pone.0119627.s007.pdf]

Sequence name	Sequence trimmed
454 adapter A1	TCCATCTCATCCCTGCGTGTCCCATCTGTTCCCTCCCTGTCTCA
454 adapter A2	TGAGACAGGGAGGGAACAGATGGGACACGCAGGGATGAGATGGA
454 adapter B1	CCTATCCCCTGTGTGCCTTGCCTATCCCCTGTTGCGTGTCTCA
454 adapter B2	TGAGACACGCAACAGGGGAAAGGCAAGGCACACAGGGGATAGG
matches found 326,555	ACGAGCGGCCA
matches found 57,840	GCCTCCCTCGCGCCATCAGCCGCGCAGGT
matches found 9,664	ATCGGTGATTAGTTGTTTCGGTCATCAAACTTCCTTCACCCAAGGG AATTTGGGGAGCCGCACAGAAACATGGTGGGTGTTACAATCTAAG TGCTGATAGGACTTGCGCTTAGTAGCTGCAGCCTAGATGAAGCGA TCTGATTCTGATAATTCACCAGTGCAGCGAAAATTACAGCGCTTTG AACCAGTGGAAAAGGCGCTATATAAATCCAAATTATTATTATTGG CCGCTCGT
matches found 7,122	ACCAAACCAGTACTAATGAAGAAAAAAAA
matches found 6,854	GAGAGATAAATGATCTAAATGTGCGTTTATTCAAAAATAGATTTTA AGCTGTGTTGATTGGGATGCTGTTTGT
matches found 7,591	TCATCTGATGATGTCAATATATCATACAAGTCATTCATTGAGAAGT TTAATGTAATGTACGAAGCGTGTTTT
